# Epileptic Colony for Virginia

**Published:** 1906-02

**Authors:** 


### Abstracts and Selections.

### Epileptic Colony for Virginia.

At a meeting of the general board of directors of the four State hospitals of Virginia, held during November, 1905, the proposition originally made eleven years ago by the able and progressive superintendent of Central State Hospital, Dr. Wm. F. Drewry, was unanimously endorsed, with enthusiasm, looking to the establishment of a much needed State institution on the industrial colony plan for epileptics. If the bill passes the Legislature, the epileptics will be removed from the three State hospitals for whites, and then the effort will be made to further develop the Industrial Colony for Epileptics, taking as many indigent epileptics as possible from the various counties, cities, etc. For many years, it has been time and again pointed out that neither the simple epileptic nor the insane is apt to be benefitted by their association, as has been so long unavoidable in the State care of these unfortunates. The general board also appointed Dr. Drewry, with three of its own members, a committee to attend the National Epileptic Association held in New York during the first week of December, 1905, to collect all the data possible bearing on the care and treatment of epileptics. —*Virginia Medical Semi-Monthly.*

P. S.—Dr. Drewry has been appointed superintendent.

